# Gauge Physics of Spin Hall Effect

**DOI:** 10.1038/srep18409

**Published:** 2015-12-22

**Authors:** Seng Ghee Tan, Mansoor B. A. Jalil, Cong Son Ho, Zhuobin Siu, Shuichi Murakami

**Affiliations:** 1 Data Storage Institute, Agency for Science, Technology and Research (A*STAR) 2 Fusionopolis Way, #08-01 DSI, Innovis, Singapore 138634; 2 Computational Nanoelectronics and Nano-device Laboratory, Electrical and Computer Engineering Department, National University of Singapore, 4 Engineering Drive 3, Singapore 117576; 3 Information Storage Materials Laboratory, Electrical and Computer Engineering Department, National University of Singapore, 4 Engineering Drive 3, Singapore 117576; 4 Department of Physics, Tokyo Institute of Technology, 2-12-1 Ookayama, Meguro-ku, Tokyo 152-8551, Japan

## Abstract

Spin Hall effect (SHE) has been discussed in the context of Kubo formulation, geometric physics, spin orbit force, and numerous semi-classical treatments. It can be confusing if the different pictures have partial or overlapping claims of contribution to the SHE. In this article, we present a gauge-theoretic, time-momentum elucidation, which provides a general SHE equation of motion, that unifies under one theoretical framework, all contributions of SHE conductivity due to the kinetic, the spin orbit force (Yang-Mills), and the geometric (Murakami-Fujita) effects. Our work puts right an ambiguity surrounding previously partial treatments involving the Kubo, semiclassical, Berry curvatures, or the spin orbit force. Our full treatment shows the Rashba 2DEG SHE conductivity to be 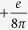 instead of −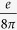, and Rashba heavy hole 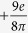 instead of −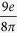. This renewed treatment suggests a need to re-derive and re-calculate previously studied SHE conductivity.

Spin Hall Effect (SHE)^1^,^2^,^3^,^4^,^5^ refers generally to the transverse separation of the electron carriers of opposite spin, quantized along the axis-z, which results in a net accumulation of spin but not charge on the left and right lateral edges of a nanoscale device. There have been many studies of the numerous possible mechanisms that could have given rise to SHE, but the gauge theory approach by Murakami *et al.*^6^ showed for the first time that in the Luttinger spin orbit coupling (SOC) system, SHE physics is related to the adiabatic alignment of electron spin with the spin orbit effective magnetic field in the momentum space. An emergent form of magnetic field, with spin quantization axis along the lab-z axis, can then be defined and linked physically to a transverse velocity component of geometric origin. Following this emergent gauge approach, SHE physics of k-geometric origin could be conveniently extended to many other systems, e.g. the linear and the cubic spin orbit in semiconductor and metal, pseudospin in massless and massive graphene, topological insulator and so forth^7^,^8^.

On the other hand, Sinova *et al.*^9^ derived the SHE conductivity for a two-dimensional-electron-gas (2DEG) system with linear Rashba SOC. Careful analysis^8^,^10^,^11^,^12^,^13^ would reveal that the SHE conductivity is in fact related to the velocity of kinetic origin. In 2010, Fujita *et al.* derived a gauge field in time (t) space that also led specifically to the kinetic velocity contributing to SHE in the 2DEG. The time-space gauge field can, in turn be linked to a t-geometric velocity which has the same form as^13^,^14^ the k-geometric velocity of Murakami. It is thus clear that one now should be particularly mindful of the multiple sources of velocity that contribute to the physics of SHE: kinetic, Murakami k-geometric, and Fujita t-geometric.

On the other hand, a separate body of work^15^,^16^,^17^,^18^,^19^ which study the spin transverse force in terms of the non-Abelian spin orbit gauge, has led to the concepts of spin orbit force and spin orbit velocity. At first glance, one might be tempted to ascribe the transverse spin orbit force to SHE. But it was soon realized that while spin orbit force might contribute to the jittering motion (Zitterbewegung) of the spin carrier, it did not quite contribute to SHE yet. In fact, it is the spin orbit velocity that provides an additional source to the SHE. This results immediately in a SHE velocity originating from an emergent gauge reminiscent of the non-Abelian Yang-Mills gauge.

We are therefore motivated to provide, in this paper, a gauge-theoretic energy framework that unifies SHE velocity of kinetic, Yang-Mills, k-geometric, and t-geometric origins for any SOC system under one equation of motion (EOM). One unified energy system that merges the two spaces of t and k is derived, debunking any previous suspicion of overlapping energy terms. The energy equation with a merged t-k identity is then used to derive the velocity equation-of-motion (EOM) for all SHE systems. Previous efforts^13^,^14^ unified Luttinger and Rashba SHE with respect to the adiabatic physics and the gauge fields, but still it remained that the Luttinger was described in k-space, and the Rashba in t-space.

## Results

The main accomplishment in our renewed treatment is that we show that a form-invariant t-k Hamiltonian is a complete energy equation, and can be used to derive a complete EOM that describes spin Hall. The complete EOM reveals in clear-cut, and non-overlapping manner, the velocity components of kinetic, Yang-Mills, and geometric origins. The significance of this result is the prediction of a reversal of sign in the SHE conductivity, suggesting the need to revisit and recalculate previously derived SHE conductivity. The Methods section will describe in details the theoretical techniques used in the process of unification. Unification provides the theoretical basis for a form-invariant, t-k manifestation of the gauge potential, which is summarized in Table 1 above.

We will now use the t-k form-invariant energy to derive a complete spin Hall EOM for the spin carrier. In the energy physics of the SOC systems, we have shown in Methods and summarized in Table 1 the unified, t-k manifestation of the local gauge. It is reasonable to ascribe before merger, the k-gauge to the k-geometric velocity due to Murakami^6^, and the t-gauge to the t-geometric velocity due to Fujita^10^. What we have done, after merger is uniting the two velocities and precluding their simultaneous manifestation. We show that a physically intuitive spin Hall EOM that encompasses the kinetic, Yang-Mills, and Murakami-Fujita velocity can be derived from the locally transformed t-k energy equation. Local transformation in this context is an abstract but useful technique to absorb the physics of spin dynamics into the gauge potential. One is free to view the effective Hamiltonian in either time or momentum space. In time space, one can define an effective magnetic field of 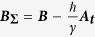. The complete SHE velocity EOM is


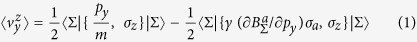



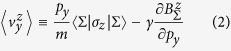


We would like to note that the velocity expression 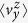 above follows from the more formal expression of 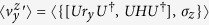, which in the case of a 2D system in lab frame leads to 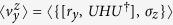 as used in Eqs. (1)&(2) above. The first term on the RHS is the kinetic velocity. The second term comprises the Yang-Mills and the Murakami-Fujita velocity as shown below


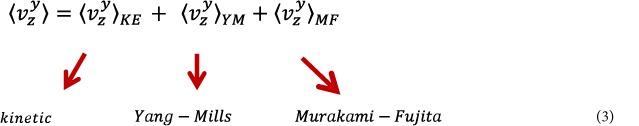


**Figure 1:**
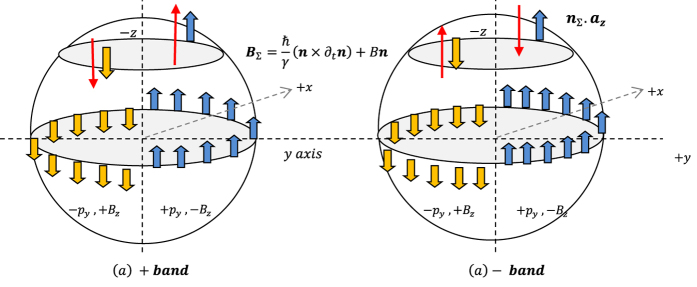
Fermi sphere of a general electron gas system in the presence of spin orbit coupling shows a distribution of the momentum, band, and effective magnetic field projected along z (B_z_). It is assumed that p = 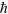
**k. The shaded region encircled by the equator shows a specific system (Rashba 2D) where for (**a**) the + band, B_z_ changes sign over the Eastern and Western hemisphere, resulting in a positive kinetic spin velocity, (**b**) the −band, B_z_ changes sign in a similar manner, thus resulting in a negative kinetic spin velocity. The slender red arrow indicates spin polarization of ±***n***_Σ_.***a***_***z***_.

The kinetic SHE velocity can also be written as


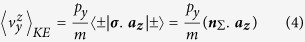


with both ***n***_Σ_ and ***a***_***z***_ having unity magnitude. The ± sign arising from the two eigenstates of 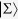 correspond to the (+) and the (−) bands, respectively. The negative sign of the spin orbit energy **i**mplies that energy is low when spin is aligned with the effective *B* field. Thus in the (+) band of Fig. 1a, spin is aligned along the *B* fields in both East and West. In the (−) band of Fig. 1b, spin is anti-aligned everywhere. The term 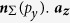 is the fractional unit of the *B* field projected to the ***a***_***z***_. Note that 
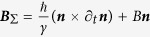
, and 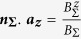, lead to 
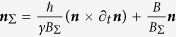
, where 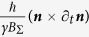 is the time**-**gauged spin orbit field, and 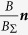 is the simple spin orbit field. The kinetic spin velocity is thus


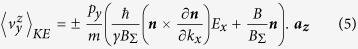


clearly showing that the external electric field is required to generate ∂_*t*_***n***. The expression 
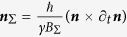
 relates to the physics of *E*_*x*_ producing an effective *B* field. For illustration in the 2D projected region surrounded by the equator, ***n***_Σ_ points along axis −z in the region of +*p*_*y*_, and axis +z in the region of −*p*_*y*_ (see illustration in Fig. 1). In other words, ***n***_Σ_ actually changes sign with *p*_*y*_. Therefore, careful examination of the (+) band (Fig. 1a) would show that *p*_*y*_***n***_Σ_.***a***_***z***_, hence 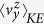 is negative in both the East and West hemisphere. The result is always positive in the (−) band. To determine SHE conductance, one needs to sum 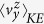 over the entire Fermi surface for both bands. Since the two band cancels one another, it is necessary to identify a region where only one band exists, or in other words, to impose a band filtering effect.

The kinetic velocity has been identified in previous time-space^10^ to contribute to SHE in the following systems, producing SHE conductivity of 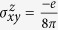 Ref. 9 in 2D RSOC hetero-structure, 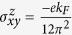
20 in n-doped cubic-Dresselhaus, and 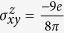
21 in Rashba heavy-hole system. There was, however, no explicit previous effort to investigate the total SHE effects that should include the Yang-Mills and the Murakami-Fujita contribution in those systems. In the Discussion section, we make explicit the other contributions to SHE in the two-dimensional Rashba SOC system, now keenly studied for technological applications in magnetic memory. The SHE velocity of Yang-Mills 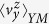 and Murakami-Fujita 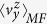 inherit their negative signs from the spin orbit energy. The explicit expression is


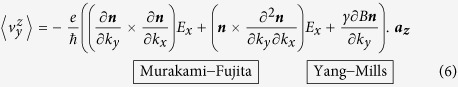


The 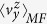 in the t-k form-invariant EOM has previously been studied in the k-space for the SOC systems of Luttinger^6^, Perel-Dresselhaus^7^,^8^, cubic-Dresselhaus in n-doped Zinc-Blende^22^. There was, however, no previous effort that explicitly investigates the effect of kinetic contribution in those systems. On the other hand, the 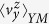 shown previously in simple (not locally gauged) quantum mechanics either vanishes for linear SOC systems, or is simply not considered. The reason for partial treatment in previous works could be due to conceptual ambiguity. It was not clear beforehand if the 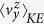 derived in the t-space gauge or the Kubo approach and the 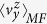 derived in the k-space gauge are strictly independent without overlapping contribution. Therefore one of the main tasks of this paper is to establish that these velocity components are not overlapping and there is no double counting. Of particular importance is the so-called 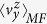 which exists in slightly different forms in both Fujita’s t-gauge and Murakami’s k-gauge pictures. This becomes clear after the form-invariant t-k Hamiltonian unifies both spaces. In unifying the two pictures, we determined that there should only be one MF velocity component. The next step is to derive all the velocity components directly from the unified or rationalized t-k Hamiltonian. The SHE velocity EOM descended from the t-k Hamiltonian shows vividly that these velocities are additive. The simple conclusions one draws here is that previous SHE conductivity related to Kubo, semiclassical, t-gauge are mostly related to 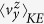. On the other hand, previous SHE conductivity related to Berry curvature or k-gauge are related to 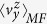. What we would like to establish therefore in this work is that future treatment of SHE should be based on a locally transformed, t-k Hamiltonian that would provide all velocity 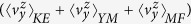 contributing to the physics of SHE.

## Discussion

**Figure 2:**
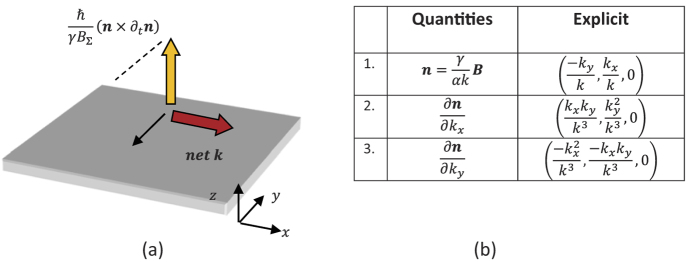
(**a**) In the case of a 2D nanostructure, where *n* lies in the *x* − *y* plane, the (*n* × ∂_*t*_*n*) term points along *z*, thus one has 
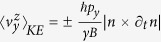
; (**b**) Table of quantities for the Rashba system that can be used to derive the SHE expression for the Rashba system.

One popular form of SHE exists in device or hetero-structure that exhibits the Rashba spin orbit coupling (RSOC). Examples are the GaAs/AlGaAs/GaAs semiconductor heterostructure, or the oxide/Metal/Pt metal multilayer, both with structural inversion asymmetry. Shown above in Fig. 2 is a schematic of the nanoscale structure where the specific RSOC exists as a result of inversion asymmetry at the interface. The effective magnetic field of the RSOC device is


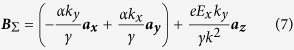


where *α* is the strength of the Rashba SOC effect.

We will first examine a two-dimensional system, where the SHE current density is obtained from the SHE kinetic velocity as follows


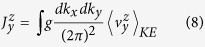


The coupling constant *g* represents spin flux for *g* =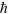/2, or electron charge flux for *g* = *e*. It has been shown that, for *g* = 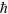/2, SHE conductivity 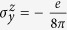 is resulted in the annular region of the Rashba bandstructure where only the (+) band exists below the Fermi energy. In the region where both (+) and (−) are below the Fermi energy, total SHE conductivity vanishes. In fact, the above has the dimension of 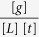, and the general SHE conductivity is 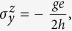 leading to respectively, the charge (*g* = *e*) and the spin (*g* =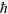/2) flux of:


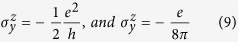


Referring to Eq. (5), we will now proceed to the Yang-Mills velocity. In the case of a linear SOC system, where effective SOC field is contained in the 2D plane, it is easy to determine that the Yang-Mills effect vanishes. We will move on to the last SHE term which is the Murakami-Fujita of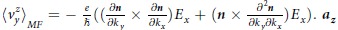, and which can in turn be broken down into two terms. The first term produces SHE current density


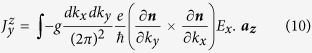


It can be shown that 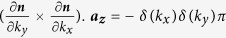. Considering that there are two bands cutting through the k = 0 point, the SHE conductivity due to 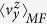 is 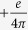. This is in addition to the 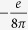 arising due to 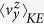 giving rise to a total SHE 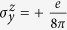. The advantage of physical clarity with the gauge theoretic approach is clearly manifest here. The first contribution to SHE conductivity originates from the kinetic velocity which is effective in the annular region of the 2D concentric circles. The second contribution originates from the Murakami-Fujita velocity that has a geometric origin, and is effective in the degenerate point where the (+) and the (−) bands intersect. The same is carried out for the Rashba heavy hole system where 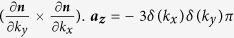. The final results are SHE 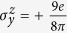 instead of the 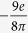 with partial treatments. Both results are summarized in Table 2.

We will make a quick remark on the second part of 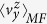, which is


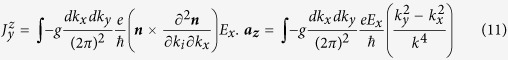


When integrated via partial fractional reformulation of the integrand, the integral delivers a “ *π*” or a “–*π*” solution depending on the sequence in which the integration is performed. But a proper treatment referring to the Fubini-Tonelli theorem leads to its vanishing results.

Therefore, the central results in this paper consist of the unified energy and the SHE EOM. On the energy, we have provided a theoretical basis to the existence of the t-k interchangeable, form-invariant Hamiltonian. This Hamiltonian allows the physics of SHE in various SOC systems to be studied under one SHE velocity EOM. The EOM descended from the t-k energy would lead to the kinetic, Yang-Mills, and Murakami-Fujita velocity which give a complete account of all contribution to SHE conductivity in any SOC system. Our work puts right an ambiguity surrounding previously partial treatments of SHE involving the use of the Kubo, semiclassical, Berry curvatures, or the spin orbit gauge. We showed for the Rashba 2DEG and the Rashba heavy hole that full treatment produces SHE conductivity of opposite signs (Table 2) due to the Murakami-Fujita contribution. More importantly, our renewed treatment can be extended to all other SOC systems to re-derive and re-calculate SHE conductivity.

## Methods

### Energy in the Unified Time-Momentum (t-k) Space

The Hamiltonian of a system with SOC can be written with the physical clarity of simple magnetism as follows:


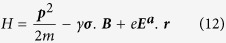


where ***B*** is a momentum dependent effective magnetic field, and *γ* has the dimension of 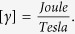 In a single-particle system with electric field, the Hamiltonian 
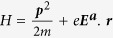
 might seem sufficient, at first glance, to describe a carrier with kinetic and potential energy. But a charge-spin carrier with a constant *p* in the presence of *E* field generates an energy term of *γ**σ***.***B***(***p***). The Dirac relativistic quantum mechanics is needed to account for this SOC energy. In the absence of any retardation effect due to scattering, the carrier would accelerate due to the *E* field. As a result, the carrier will acquire an *E*-dependent energy 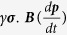. This is a geometric related energy that can only be revealed with the local gauge transformation or time-dependent perturbation treatment.

### Momentum Space

The approach that has been used to derive SHE velocity in refs 6,22 is based on a local gauge transformation in the k-space. In the “Schrodinger” picture, transformation applies to the k-space only, but not the t-space, because momentum is time-independent. Local transformation leads to


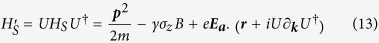


where the gauge potential 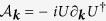, with dimension 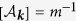, is associated with the SHE velocity via the Karplus-Luttinger method.

### Time Space

The t-space approach has on the other hand, been adopted in refs 10, 11, 12, 13 to derive SHE in refs 9,20,21. To study the transformation in the fourth time space, an “Interaction” picture is necessary. The term *γ**σ***.***B***(***k***) would become *γ**σ***.***B***(*t*), where 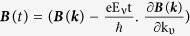 is the instantaneous SOC field. One needs to split the Hamiltonian into two parts, i.e. *H*_*S*_ = *H*_0_ + *V*_*S*_, and note the following:


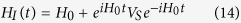


or *H*_*I*_(*t*) = *H*_0_ + *V*_*I*_(*t*). In the “Interaction” picture, one has *i*
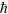
∂_*t*_*ψ*_*I*_(*t*) = *V*_*I*_(*t*)*ψ*_*I*_(*t*). Thus a transformation in time space is appropriate here


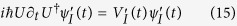


where 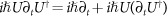, and the second term is contingent upon ∂***k***/∂*t* ≠ 0, a condition that would be fulfilled when *E* field is present in the device. We assign as follows: 
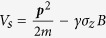
, and *H*_0_ = *E*.*r*. On the RHS of Eq. (5), 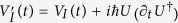, where *i*
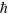
U(∂_*t*_*U*^†^) is also known as the time-space gauge with the dimension of energy. The unitary rotation operator *U* in local space is


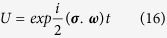


and one can quickly obtain that 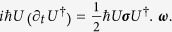 Rearranging, one can now write the locally time-transformed Hamiltonian in “Interaction” picture as


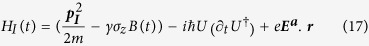


Equation (17) in time space has the same form as Equation (13) in momentum space. Re-examining the time-gauge in the k-space,


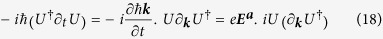


leads to one obtaining a gauge expression in the k-space. Note that 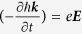. The fact that *H*_0_ = *e**E***^***a***^.***r*** is important for the survival of the gauge 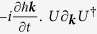 for the gauge coupling constant is only non-vanishing because of *H*_0_ = *e**E***^***a***^.***r***. Inverse transformation of 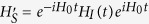 would lead to higher order terms with respect to −*i*
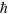
*U*(∂_*t*_*U*^†^). Dropping the higher order terms, the following is arrived


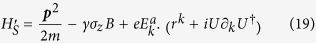


Note that for expression *iU*∂_*k*_*U*^†^, subscripts are subject to 
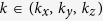
, while for expression *r*^*k*^, subscripts are subject to 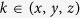. Equation (19) is transformed in the t-space at the outset, but now appears identical to the k-space Equation (13). The energy equations have thus been merged under a form-invariant t-k identity. What is clear from the above is that the gauge potential derived, interchangeably in the k-space or the t-space, will not exist simultaneously in both spaces. It is now logical to conclude that the gauge potential has an independent, non-overlapping contribution to the geometric velocity of SHE. Back to the Lab frame (mere relabeling of the axis), one can write the Hamiltonian in the form of Zeeman magnetic field as in Eq. 20(a), and in the form of Lorentz magnetic field as in Eq. 20(b),


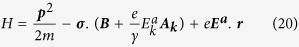



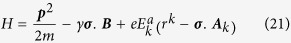


Note that in the above the convention 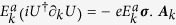 is followed. Similar convention in time space is followed 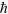
(*iU*^†^∂_*t*_*U*) = −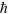
***σ***.***A***_*t*_.

We would, however, note that the Hamiltonian alone, pre- or post-transformed could not reflect the full physical reality of the 2D system in which electron propagates in time. As electron propagates in momentum and spin space, so does the spin-orbit magnetic field, and the actual physics related to such effect can only be reflected in the wave-function that is solved taking into account the path of evolution. This is a separate task that needs to take into account approximations therein. Simply stated, a stationary Hamiltonian cannot reveal how electron propagates in time, as it only reveals the stationary eigen-states, which has spin aligned parallel or anti-parallel. Local gauge transformation locks the spin orbit field to a specific axis (e.g., *z*), generating a gauge field. Still, the post-transformed Hamiltonian only reveals the stationary eigen-states, but one which is more intuitive now with spin aligned to a total field out of plane. Note however, that in both pre- and post-transformed Hamiltonian, expectation values of observables remain the same. But this is not important as we are not interested in the stationary expectation values. What we need from these Hamiltonians are clues to determining the actual propagation physics of electron. In previous works^2^,^3^,^6^, adiabatic approximation of spin locking to spin orbit field is commonly applied. Parallel spin locking to the spin orbit field can then be associated with a scalar gauge field that is physical, while anti-parallel locking produces a similar but negative effect. Initial spin state determines the proportion of parallel or anti-parallel alignment. In this paper, adiabatic approximation of spin locking to the total field instead of the in-plane spin orbit field is considered. In SHE, parallel and anti-parallel spin locking will be equally likely, and this is true for initial spin state that is random or in-plane. SHE will then be the *z*-projection of the total field taken along the two lateral sides of the device.

## Additional Information

**How to cite this article**: Tan, S. G. *et al.* Gauge Physics of Spin Hall Effect. *Sci. Rep.* **5**, 18409; doi: 10.1038/srep18409 (2015).

## Figures and Tables

**Table 1:** Summary of important gauge theoretic quantities expressed in both time and momentum spaces.

		Time-space	Momentum-space
1	Local gauge transformation and vector potential notation	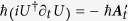	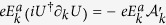
2	Physics of effective magnetic field 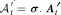 , 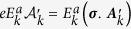	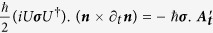	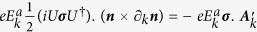
3	Hamiltonian in the locally rotated frame	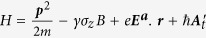 Rotation of the *z*–axis to *B*(*t*)	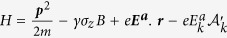 Rotation of the *z*–axis to 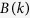
4	Hamiltonian in lab frame, showing effective magnetic fields	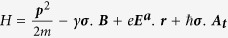	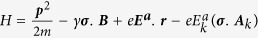 ***k*** in three-momentum space

**Table 2:** The gauge theoretic physics provides a full treatment of the SHE, showing results opposite in sign to previous treatments, summarized above.

	SHE Conductivity	New SHE Conductivity
Rashba 2DEG	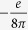	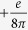
Rashba Heavy hole	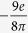	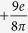
